# Hyaluronic acid‐rich burger separator edible disc prepared from slaughterhouse waste

**DOI:** 10.1002/fsn3.2740

**Published:** 2022-01-20

**Authors:** Mahboubeh Kalantarmahdavi, Amir Salari, Zahra Pasdar, Mohamad Reza Amiryousefi

**Affiliations:** ^1^ Department of Food Hygiene and Aquaculture Faculty of Veterinary Medicine Ferdowsi University of Mashhad (FUM) Mashhad Iran; ^2^ 1019 School of medicine, medical science and nutrition University of Aberdeen Aberdeen UK; ^3^ Department of Food Science and Technology Neyshabur University of Medical Sciences Neyshabur Iran

**Keywords:** bovine bone gelatin, chicken feet, edible film, hyaluronic acid, ovine muscle fascia

## Abstract

In this study, the edible films from chicken feet (CF), ovine muscle fascia (MF), and bovine bone gelatin (Gel) were prepared and their characteristics were analyzed, and we also evaluated the sensory quality of raw and cooked hamburgers using the edible films. The quantities of the CF and MF hyaluronic acid were evaluated using colorimetry and spectrophotometry. The CF, MF, and Gel films were prepared by solvent casting method. Results indicated that the concentration of hyaluronic acid in CF (124.11 ppm) was greater than MF (101.11 ppm). The antioxidative property of the CF film (18.47%) was greater than the Gel (1.88%) and MF (Undetectable) film. The CF film was more resistant to water vapor permeability (2.75 × 10^–9^ g/m.s.pa) than the MF (1.57 × 10^–8^ g/m.s.pa) and Gel (1.5 × 10^–7^ g/m.s.pa) films. The Gel film had more appropriate mechanical properties than CF and MF films. The films kept burgers patties independent from one another and prevented them from sticking and freezing together. MF and CF films were able to promote the organoleptic properties of raw and cooked hamburgers in taste and texture.

## Graphical abstract

Slaughterhouse by‐products have high nutritional value and are a rich source of hyaluronic acid. In this study, the edible films from chicken feet (CF), ovine muscle fascia (MF), and bovine bone gelatin (Gel) were prepared and their characteristics were analyzed, and we also evaluated the sensory quality of raw and cooked hamburgers using the edible films.

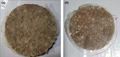

## 1 INTRODUCTION

Food packaging applications have made major use of biopolymers as film‐forming materials. From the past, proteins and polysaccharides have been the most extensively experimented materials for film or coating processing. Nowadays, a variety of naturally derived polymers are accessible for application in the form of biomaterials. Examples include polysaccharides derived from plants, animals, fungi, and bacteria, as well as proteins, lipids/surfactants, and other polymers (Yu, 2009). Importantly, many of these polymers are extracted from sources that could be used for human consumption. Therefore, the ability to employ other substitute sources from agroindustrial residues, such as waste materials, may serve as a more economical method for recycling these waste products into valuable products. Such an example includes bio‐derived polymers, nutritional, and low‐cost raw materials, which can be found in agroindustrial residues.

Remains of carcasses, butcher and slaughterhouse waste, blood, feathers, wool, hides and skins, and fallen stock represent the major animal byproducts acquired from the animal processing industry. The quantity of animal byproducts in the animal industry typically surpasses 50% of the live weight, and the dressing percentages of carcasses range from 57% (standard cattle grades) to 70% (chicken), depending on the animal. Key polymers derived from animal bones, skins, and feathers include collagen, gelatin, and keratin, respectively (Ferreira et al., 2016).

Poultry and sheep (ovine) production constitutes one of the most important industries in Iran. Slaughterhouses, like most food industries, produce waste that needs to be managed consistently. According to FAO, 1000 head per unit of chickens were produced in Iran during 2019 and 25% of this amount was waste (Food and Agriculture Organization of the united nation, 2019). In the endeavor to achieve greater sustainability, investing in low‐cost productivity support methods is essential. In addition, it is important to focus on the use of by‐products in the poultry industry. Chicken feet are comprised of 85% protein, most of which is mainly collagen, and 2.7% fat (Almeida & Lannes, 2013). The chicken feet collagen primarily comprises of type I and type II collagen and also contains 11.7% praline, 30% glycine, and 12.7% alanine and glutamic acid (Liu et al., 2001). According to Lee et al. (2015), chicken feet protein film (3:2 ratio (w/w) of glycerol–sorbitol) had tensile strength (TS) of 7.13 MPa and elongation of 21.78% (Lee et al., 2015). Based on these characteristics, the extraction of chicken feet seems to be an appropriate material for making edible films.

In mammals, collagen encompasses 25%–30% of the total body protein content. Collagen is also an important constituent of muscle tissue (forming 1%–2% of muscle tissue) where it is a chief component of the endomysium (Silvipriya et al., 2015). Moreover, fascia, which is a continuous viscoelastic tissue synthesized from layers of dense connective tissue (collagen types I and III) and interfaced by loose connective tissue, has a prominent viscoelastic property (Cowman, Schmidt, Raghavan & Stecco, 2015). Deep fasciae can be divided into two major categories: the epimysial fasciae and the aponeurotic fascia (Fede et al., 2018). The epimysial fasciae consists of all the connective tissues which surround and interpenetrate the muscles and tendon, and are tightly adherent to them, such as epimysium, perimysium, and endomysium. The aponeurotic fascia are fibrous connective tissue layers that cover muscles and connect different segments at a distance (Fede et al., 2018). This tissue is one of the by‐products of discarded sheep muscles. So far, waste from various animal products has been used to produce edible films, but in this study, for the first time, aponurotic fascia extract was used as a rich source of collagen and hyaluronic acid to prepare an edible film.

In 1934, a new glycosaminoglycan (GAG) extracted from bovine vitreous was described by Hekarl Meyer and John Palmer. They found that it contained uronic acid and an amino sugar but no sulfur ester. They suggested the name “hyaluronic acid,” which is derived from the combination of the words hyaluronic acid (vitreous) and uronic acid. This material is one of nature's multifunctional and intriguing macromolecules and is often called “hyaluronan.” It is a vital component of the extracellular matrices (ECMs) in most mature tissues in all vertebrates. Hyaluronan is considered as a biologically suitable material for use in biodegradable polymers in the food packaging industry due to its water‐holding capacity and high viscoelasticity. Moreover, it is biocompatible and biodegradable (Lewandowska et al., 2017; Pan et al., 2020). The cost of hyaluronic acid products and their derivatives is high and this ranges from US $2000 to $60,000 kg^−1^ (Pires et al., 2010). Due to hyaluronic acid's high price, it can be obtained from different sources to minimize its cost and improve films’ physicochemical properties. Chicken and ovine by‐products, such as chicken feet and ovine muscle fasciae, have considerable amounts of collagen and hyaluronic acids (Hashim et al., 2014).

The incomplete hydrolysis of collagen during moist heating produces gelatin, and the use of gelatin films for food preservation applications such as in coatings or films has been broadly researched (Bergo et al., 2013). Despite the very hydrophilic nature of gelatin, gelatin films have been demonstrated to have very good processability and they possess appropriate barrier and mechanical properties. Moreover, the origin of the gelatin and its film‐processing features have a substantial impact on the operational features of the ensuing gelatin‐based films (Gómez‐Guillén et al., 2009).

In the commercialization of burger products, the breaking of slices during separation just before consumption is one of the major problems. Commonly, slice separator films are used to avoid sticking, such as oriented polypropylene, PET, or paper coated with PVDC dispersions or PE (Schneider et al., 2010,). Slice separator edible films could be a good alternative to commercial separators due to the lack of negative interactions with the product and the probable good acceptance of consumers; however, the information about the application of edible films or coatings as separator layers is very limited (Cruz‐Diaz et al., 2019). The aim of this study was to prepare films utilizing extracts of chicken feet and ovine muscle fascia to determine the content of hyaluronic acid in them. In addition, the physicochemical and mechanical properties of CF and MF films were evaluated and were compared with the properties of bovine bone gelatin film. Their potential as burger slice separator films during freezing was assessed and sensory analysis was also performed.

## 2 MATERIALS AND METHODS

### 2.1 Sample preparation

Frozen chicken (male) feet and ovine (male) muscle fascia were purchased from a slaughterhouse. The samples were immediately transferred to the laboratory and washed thoroughly to remove any contaminations, and later chopped into 3–5 cm^2^ pieces. These pieces were then stored in a freezer (−20°C) separately. Gelatin powder made from bovine bone was also purchased.

### 2.2 Extraction

For extract preparation, the CF and MF samples were defrosted at 4°C–5°C. Approximately 1000 g of CF and MF was combined with 5000 ml of water separately, and then boiled for 5 h. Thereafter, the extractions were filtered, autoclaved at 121°C for 20 min, and then cooled at room temperature (23°C).

### 2.3 Determination of hyaluronic acid concentration

#### 2.3.1 Standards and sample preparation

According to the method by Dong et al. (2014), approximately 50 mg of analytical grade D‐glucuronic acid (Merck KGaA) was dissolved in distilled water of 100 ml volume. To obtain 500 μg/mL of reference solution, the solution was diluted 10‐fold. Water was added to varying volumes of 0.0, 0.2, 0.4, 0.6, 0.8, and 1.0 ml of the reference solution in topped test tubes, resulting in a final volume of 1.0 ml. The test tubes were then cooled to 4°C by being placed in ice water. Approximately 5 ml of freshly prepared sodium sulfate with sodium borate (Merck KGaA) was added to each test tube (4.77 g disodium tetraborate of analytical grade in 500.0 ml sulfuric acid of high‐grade purity). The test tubes were sealed with caps and subsequently shaken, and then heated in a boiling water bath for 10 min. Thereafter, they were cooled to room temperature and 1.25 ml of carbazole (Merck KGaA) solution (0.125 g of carbazole in 100.0 ml of absolute alcohol) was added to each test tube. The test tubes were resealed, shaken, and heated again for 15 min in a hot water bath (100°C). Later, they were cooled down in ambient condition, and the color of the solution changed to purple. We measured the absorbance of solutions at 530 nm (Spectrophotometers‐UV‐Visible, Mecasys) against a blank sample. The hyaluronic acid concentration could then be determined based on the standard calibration curve and the dilution ratio (Dong et al., 2014; Amiryousefi et al., 2016).

### 2.4 Film formation

The films were prepared by using a casting method: 100 ml of the CF’s and MF’s liquid extract was mixed with the concentration of glycerol as plasticizer (5% w/v). For the preparation of gelatin film, 10 g of dry matter and 1 g of glycerol were dissolved in 100 ml of boiling water. Approximately 10 ml of each film solution was poured into an 8 cm Petri dish and dried at 37°C for 18 h. The dried films were placed in plastic zipper bags and stored in the refrigerator prior to testing.

### 2.5 Characteristics of the films

#### 2.5.1 Film thickness

The thickness of the films was measured using a digital micrometer (Mitutoyo No.293‐766) with exactness of 1 μm at 10 random positions on the film. The obtained thickness values were applied for calculating the films’ water vapor permeability and tensile properties.

#### 2.5.2 Moisture content

The moisture content of the films was measured by drying the samples in an oven at 105°C until the weight remained constant. The weight loss of samples was determined before and after drying with a scale accuracy of 0.001 g (Khodaei et al., 2020).

#### 2.5.3 Solubility in water

Using the method outlined by Tongdeesoontorn et al. (2012), the water solubility of the films was determined. Pieces of the films (3cm × 3cm) were dried in an oven (105°C for 5 h). Then, the films were placed in a beaker with 30 ml distilled water and they were shaken for 24 h at 25°C. The undissolved remnants were filtered and dried at 105°C for 5 h (Tongdeesoontorn et al., 2012).

#### 2.5.4 Water vapor permeability (WVP)

In line with the ASTM E96‐00 method, we determined the WVP of films gravimetrically. The film samples with effective area of 31.4 mm^2^ were situated on test cups which each contained 3.0 g of anhydrous sodium chloride (0% relative humidity, RH, assay cup) and were then sealed. Each cup was placed in a desiccator containing a saturated solution of sodium chloride at 25°C. The weight of the cups was measured throughout 3 h intervals for 48 h (ASTM International, 1998). The WVP was calculated using the following formula:

$$\text{WVP}=\left(\Delta m/\Delta t\;A\right).\;\left(X/\Delta P\right)$$

where (*A*) represents the area of exposed film surface in m^2^, (Δ*m*/Δ*t*) is the weight of moisture gain per unit of time (g/s), (*X*) represents film thickness m, and (Δ*p*) is the difference in water vapor pressure between two films.

#### 2.5.5 Contact angle measurements

In line with Beigomi et al. (2018), the wetting characteristics of the films were evaluated by measuring the contact angle. We utilized the sessile drop method to measure the contact angle. This involves an optical contact measuring device (OCA20, Data Physics, GmbH) supplied with a CCD camera, and an automatized syringe control system. To measure the contact angle, which is the angle the liquid creates with the solid when it is deposited on it, an image analysis software tool (SCA20) was utilized. To carry out the measurements, a drop of distilled water from a syringe was placed on the film surface, with dimensions measuring 4.0 mm × 4.0 mm. Up to 10 measurements were taken for each film type on different areas of the surface of the film, and mean values were determined. All measurements were taken in an open air environment with room temperature and a relative humidity of 35% ± 5% RH (Beigomi et al., 2018).

#### 2.5.6 Tensile strength (TS) and elongation at break (EB)

To evaluate the mechanical properties of the films, we utilized their tensile strength (TE) and elongation at break (EAB) as proxy measures. These mechanical qualities were evaluated at 25°C and 50% RH. In this study, we utilized the D882‐18 standard test method, and the H5KS Stable Micro System, UK, was used as the testing instrument. The films were conditioned in 50% RH in a desiccator containing saturated solutions of Mg(NO_3_)_2_ for 48 h. Film samples, 20 mm × 100 mm, were cut from each film and were located between the grips of the testing instrument. The initial grip distance and the cross‐head speed were set at 50 mm and 5 mm/min, respectively (ASTM International, 2012).

#### 2.5.7 Scanning electron microscopy

To determine the films' microstructure, we utilized Scanning Electron Microscopy SEM (EM‐3200, KYKY). The films were frozen in liquid nitrogen and fractured. Thereafter, they were mounted onto aluminum stubs with double‐sided tape, and then were coated with a thin layer of gold using a BAL‐TEC SCD 005 sputter coater (BALTEC AG, Balzers, Liechtenstein). Low pressure and an accelerating voltage at 20 kV were used for *SEM* imaging.

#### 2.5.8 FTIR spectra of the films

The infrared spectrum of absorption or emission of a matter was determined using Fourier transform infrared spectroscopy (FTIR) (Thermo Nicolet, Avatar 370). At first, the films were powdered to set up the discs, and then 70 mg of spectroscopic grade KBr was mixed fully with roughly 2 mg of the film's powder. Subsequently, the powder was hard‐pressed into pellets to obtain a transparent disc with 15 mm in diameter and 0.54 mm thickness. The FTIR spectra were obtained in the 4000–400 cm^−1^ range, at 25°C, by co‐adding 32 scans with 4 cm^−1^ spectral resolution.

#### 2.5.9 Differential scanning calorimeter (DSC)

The thermal properties of films were analyzed with Mettler Toledo, DSC‐1, Switzerland. Samples (approx. 7 mg) were weighed into the reference (an empty aluminum pan), and heating rate was programmed by setting the heater at 10°C/min between a range of −100°C and 200°C. Glass transition temperatures (Tg) and melting temperatures (Tm) of each film were established from the resulting thermo grams as the midpoint temperature of the shift in the baseline due to the change in the heat capacity upon the glass transition. Readings of Tg were taken twice and the average of the results has been presented.

#### 2.5.10 Radical scavenging activity of the films

DPPH radical scavenging activity was carried out according to the procedure of Brand‐Williams et al. (1995) (Brand‐Williams et al., 1995). Twenty‐five milligram of the sample was dissolved in 5 ml of distilled water. Later, 0.1 ml of the solution was mixed with 3.9 ml of the DPPH solution (0.1 mM methanol solution). Then, they were incubated in the dark for 30 min. at 25°C. While mixing the DPPH, a stable nonradical form of DPPH was obtained with simultaneous change in the violet color to a pale yellow. The Perkin‐Elmer spectrophotometer was used to measure absorbance at 517 nm. We determined the percentage of DPPH radical scavenging activity using the subsequent equation:

$$\begin{array}{c} \text{Radical}\;\text{scavenging}\;\text{activity}\;\left(\%\right)\\ =\left(\left(A\;\text{reference}\;‐\;A\;\text{sample}\right)/A\;\text{reference}\right)\times 100 \end{array}$$

### 2.6 Sensory analysis

Burgers (50 g) were prepared manually with a round‐shaped mold. Burgers were separated with films aseptically at room temperature. Fresh burgers without films were used as controls. All samples were placed in trays covered with aluminum foil and stored at −20°C until analysis. Sensory analysis was performed by a group of five trained panelists using a 5‐point hedonic scale ranging from “very strong like, score 5” to “very strong dislike, score 1.” Score of 1–5 was assigned for the overall acceptability of the cooked samples (control and wrapped), which was determined by assessing the appearance, color, odor, taste, texture, and flavor (Stone and Sidel, 2004).

### 2.7 Statistical analysis

All tests were performed three or more times. We have presented the data as mean values with their standard deviation and these values were used in the statistical analysis. The significant differences were found by one‐way ANOVA and the means were compared using Duncan's multiple‐range test *p* < .05. We used statistical software SPSS (Inc., Ver. 21) to carry out the statistical analysis.

## 3 RESULTS AND DISCUSSION

### 3.1 Determination of hyaluronic acid concentration

**FIGURE 1:**
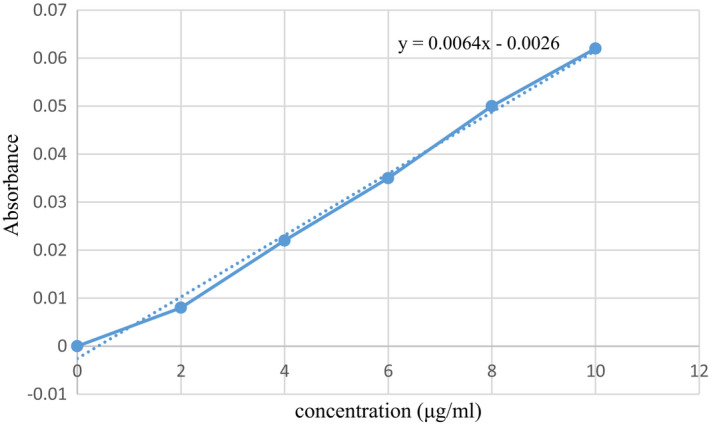
Reference concentration of hyaluronic acid measured with a spectrophotometer

**FIGURE 2:**
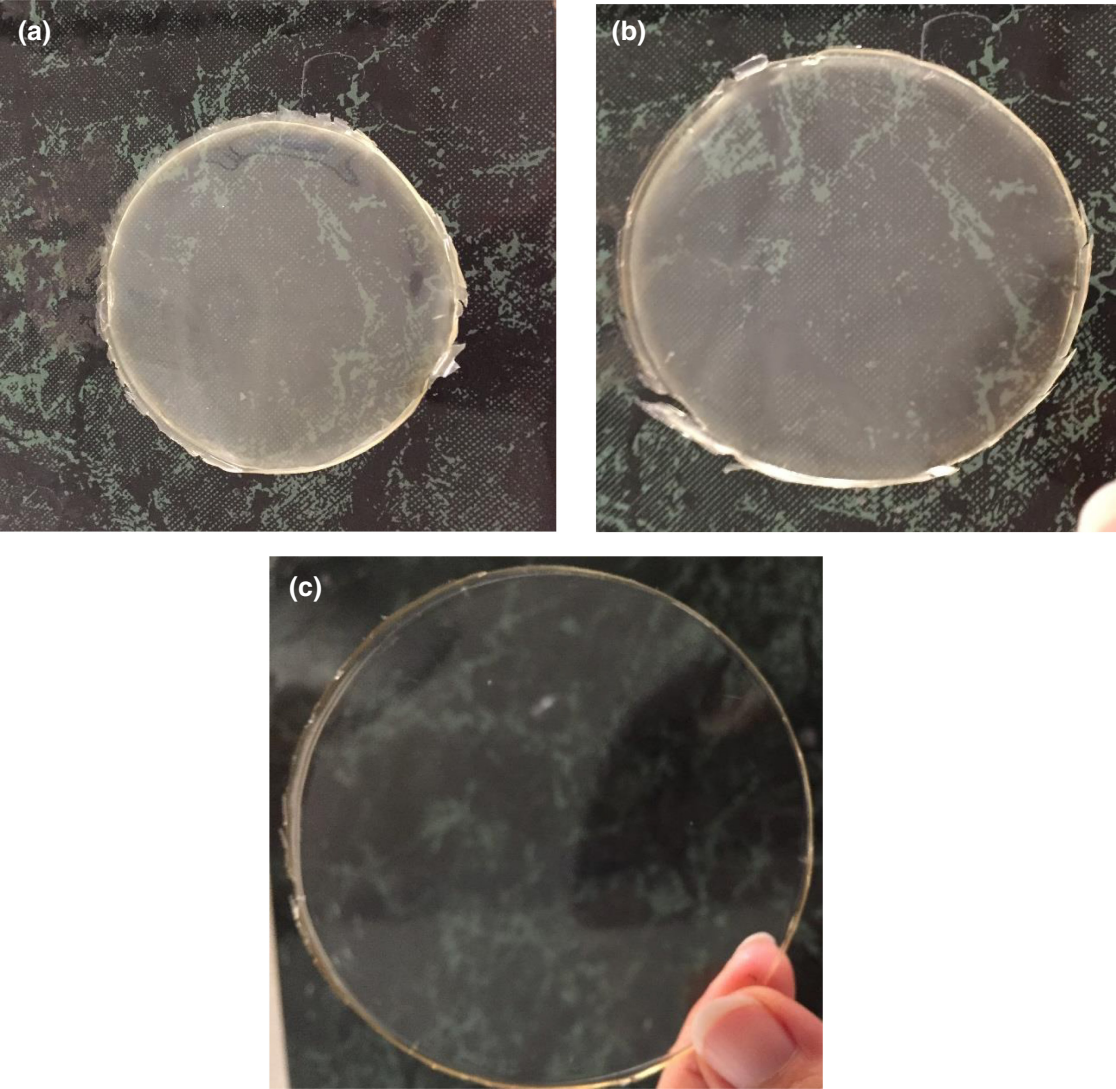
Images of films, chicken feet film (a), ovine muscle fascia film (b), and bovine bone gelatin film (c)

Figure 1 shows the spectra of the hyaluronic acid standards. Based on the equation obtained from the standard diagram, the amount of hyaluronic acid in CF extract was 124.11 and 101.40 ppm in the extract obtained from MF, respectively. The data show that the amount of hyaluronic acid in CF extract is significantly higher than in the MF extract. According to previous studies, rooster comb has the highest amount of hyaluronic acid in living tissues. The amount of this substance in rooster comb is about 7500 μg/ml (Kanchwala et al., 2005). This amount is about 60 times more than the hyaluronic acid in CF. However, hyaluronic acid in the rooster comb is complex with proteoglycans, which makes extraction of high purity difficult and costly (Hanani et al., 2019). Recently, *Streptococcus* species are used for microbial production of hyaluronic acid. In the study by Al‐Saadiaa et al. (2016), hyaluronic acid was extracted from *Streptococcus pyogenes*, 67.9 ng/ml at 7.5 pH (Al‐Saadiaa et al., 2016). However, genetic mutations in this genus of bacteria and the possibility of producing toxins have limited their application (Li et al., 2020). Nevertheless, the use of poultry and sheep by‐products is not only inexpensive and more accessible than many sources but also has a significant amount of hyaluronic acid, which can be used in food and packaging industries. The extracts of CF and MF generated in this work could simply result in homogeneous filmogenic solutions, which provides translucent films that can be readily manipulated (Figure 2).

### 3.2 Thickness

As can be seen in Table 1, the average thickness for CF and MF films is 0.10 mm. While the thickness of Gel film is 0.17 mm, it is significantly larger than the other films. Hanani et al. (2019) reported that the thickness of fish gelatin film is 0.06 mm and Li et al. (2020) reported that the B type gelatin film is 0.07 mm (Hanani et al., 2019; Li et al., 2020). As such, these materials were thin enough to be considered as films, and the effect of the observed differences in thickness on the film's functional properties could be negligible. Controlling the film thickness is an essential factor which may influence mechanical, barrier, and transparency properties of the films (Ghanbarzadeh & Almasi, 2011). Moreover, discrepancy in the film thickness may result from the type of solid matter used as well as its amount; the film preparatory methods and drying conditions may also influence the thickness (Galus & Lenart, 2013).

**TABLE 1:** Physical properties of the films

Film	Thickness (mm)	MC (%)	WS (%)	WVP (g/m.s.pa)	Water contact angle (degree)
CF	0.10 ± 0.02^b^	7.40 ± 0.50^a^	68.49 ± 0.06^b^	2.75 × 10^–9^ ± 0.08^a^	90.209 ± 0.91^b^
MF	0.10 ± 0.03^b^	5.90 ± 0.08^b^	99.08 ± 0.00^a^	1.57 × 10^–8^ ± 0.08^b^	92.27 ± 0.23^a^
Gel	0.17 ± 0.05^a^	7.45 ± 0.02^a^	40.65 ± 0.05^c^	1.5 × 10^–7^ ± 0.10^c^	79.2 ± 0.6^c^

Note

Different superscript letters in the same column indicate significant differences among formulations (*p* < .05). Values are means of three replicates ± standard deviation.

Abbreviations: CF, Chicken feet film; Gel, Bovine bone gelatin film; MC, moisture content; MF, muscle fascia film; WS, water solubility; WVP, water vapor permeability.

### 3.3 Moisture content

According to the data presented in Table 1, a significant difference was found between the average moisture content of the CF and Gel films (7.40% and 7.45%, respectively) and the MF film (5.90%). The moisture content of chicken feet is 65.08% (Hashim et al., 2014). Lee et al. (2015) also reported that the moisture content of chicken feet protein film with glycerol and sorbitol as plasticizer is 10.17% and Hanani et al. (2019) reported that the moisture content of fish gelatin film is 11.05% (Hanani et al., 2019; Lee et al., 2015). The higher humidity in CF and Gel films is dependent on its hydrophilic nature which is mostly due to the tendency to form hydrogen bonds with water molecules. The reduction in moisture content is mainly related to the hydrophobicity of the fats present in the film components with gelatin base, which greatly reduces the film water absorption capacity (Li et al., 2020).

### 3.4 Solubility in water

The films solubility percentage after a 24‐h immersion in water is shown in Table 1. This shows to be 99.09% for the MF film, 68.49% for the CF film, and 41.62% for Gel film. After 24 h of incubation in water, the CF and MF films changed shape while the gelatin film completely retained its structure. Specifically, the solubility of MF films (99.08%) was significantly higher (*p* < .05) and the solubility of Gel film (40.65%) was significantly lower (*p* < .05) than the other films. In one study, the film solubility level for chicken skin gelatin was 94% (Loo & Sarbon, 2020), and in another, the solubility of fish skin gelatin film was 68.64% (Hanani et al., 2019). Moisture content, which depends on the wettability and free surface energy, increases the films solubility which is one of the major advantages of the films (Loo & Sarbon, 2020). Polypeptides cross‐linkages and higher molecular weights in gelatin result in lower water solubility compared to muscle fascia and chicken feet. Lower water solubility of the films due to lower water activity and thus less possible contamination in the presence of water are desirable characteristics for food packaging (Escamilla‐García et al., 2019).

### 3.5 Water vapor permeability (WVP)

Table 1 shows the WVP of films. According to the results, the CF film had significantly lower WVP (2.75 × 10^−9^ g/ m. s. Pa) than the MF film (1.57 × 10^–8^ g/ m. s. Pa) and the Gel film (1.5 × 10^–7^ g/ m. s. Pa). These data are consistent with findings by Lee et al. (2015), where they reported the WVP of chicken feet protein film without plasticizer to be 3.44 × 10^–9^ g m/m^2^ s Pa (Lee et al., 2015). In another study, Li et al. (2020) report that the WVP of gelatin (type B) film is 8.83 × 10^−11^ g/s. Pa (Li et al., 2020). According to Hashim et al. (2014), the fat content of chicken feet is 3.9% (Hashim et al., 2014). The presence of lipids in the film structure resulted in a decrease in the WVP. The small fat particles lead to their homogeneous distribution in the film matrix, which can reduce WVP (Pérez‐Gago & Krochta, 2001). Also, the incorporation of hydrophobic components (e.g., essential oils and plasticizers) caused the reduction in WVP due to the increased hydrophobicity of biopolymer‐based films (Almasi et al., 2020). Lower WVP values are preferred to reduce unacceptable alterations in product quality (Orozco‐Parra et al., 2020).

### 3.6 Contact angle measurements

The contact angle is one of the most frequently used measures when assessing surface properties of biopolymers. It provides information regarding films’ surface wetting or nonwetting properties. The results have been demonstrated in Table 1. There is a significantly lower (*p* <.05) contact angle for the Gel film (79.20°) as opposed to the MF film (92.27°) and CF film (90.209°). The contact angle of a water droplet on a surface relates to the surface's hydrophobicity (Giovambattista et al., 2007). Bracco and Holst indicate that hydrophobic (nonwet table) surfaces have contact angles larger than 90°, and hydrophilic surfaces (wet table property) tend to contact angles below 90° (Bracco et al., 2013). Accordingly, the two films of CF and MF were hydrophobic, but the Gel film was hydrophilic. As shown in Table 1, the surface hydrophobicity for the CF and MF films was low but they are hydrophobic films. A similar observation for the Gel film has been reported where the contact angle was found for B type gelatin film (74.2°; Li et al., 2020). A possible explanation for the results could be related to the fat content of the films, which would correlate with their hydrophobic qualities. For functional uses including food packaging, it is important that edible films have a low affinity to water.

### 3.7 Tensile strength (TS) and elongation at break (EB)

TS and EAB of the films are demonstrated in Table 2. The maximum tensile stress a film can sustain corresponds to its TS, whereas the maximum change in length of a test specimen before breaking is indicated by reflects its EAB is (Pereda et al., 2012). Mechanical strength is a general necessity to retain the integrity of packaging films and to be able to withstand external stress (Yang & Paulson, 2000). The TS of the Gel film (5.7 MPa) was significantly greater (*p* <.05) than the MF (3.9 MPa) and CF (2.49 MPa) films. Lee et al. (2015) reported that the TS of chicken protein film with sorbitol was 3.38 MPa and Hanani et al. (2019) reported that the TS of a pure fish gelatin film was 7.22 MPa (Hanani et al., 2019; Lee et al., 2015). The strength and weakness of the hydrogen bonds of the film molecules determine the TS, so the stronger the internal network and the cohesion of the film, the higher the TS. The increase in TS may also be due to differences in molecular weight, size, number of oxygen atoms, and hydrophilicity of the film matrix molecules.

**TABLE 2:** Antioxidant activity of the films—DPPH method, mechanical, and thermal properties of films

Film	Radical scavenging activity (%)	Tg (ºC)	Tm (ºC)	TS (Mpa)	EAB (%)
CF	18.47^a^	33.80 ± 0.81^b^	35.42 ± 1.91^b^	2.49 ± 0.15^c^	89.05 ± 1.08^a^
MF	Undetectable^c^	28.54 ± 0.28^c^	30.36 ± 1.55^c^	3.9 ± 0.43^b^	85.25 ± 0.90^b^
Gel	1.88^b^	38.55 ± 0.1^a^	40.53 ± 0.55^a^	5.7 ± 0.51^a^	70.50 ± 1.70^c^

Note

All measurements were performed at 25°C and RH = 50%. Different superscript letters in the same column indicate significant difference (*p* < .05).

Abbreviations: EAB, elongation at break; Tg, glass transition; Tm, melting temperature; TS, tensile strength.

Among the different films, the CF film had a greater EAB (89.05%) compared to the MF (85.25%) and Gel (70.50%) films, respectively. In contrast to the TS, the EAB of CF film increased significantly, implying that the film was more pliable when compared to the gelatin film. The occurrence of lipid in CF films resulted in increased EAB of films being observed (Table 2). It appears as though the presence of lipid globules throughout the film matrix leads to a decrease in continuity and interconnection of the protein network. Moreover, variability in molecular weight, hydrophilicity, and number and size of oxygen atoms of the CF extract might be a result of the greater EAB. Thus, interruption in continuity in microstructure of the film caused by the occurrence of lipid globules may impact the film's ability to stretch (Wang et al., 2009).

### 3.8 Scanning electron microscopy

**FIGURE 3:**
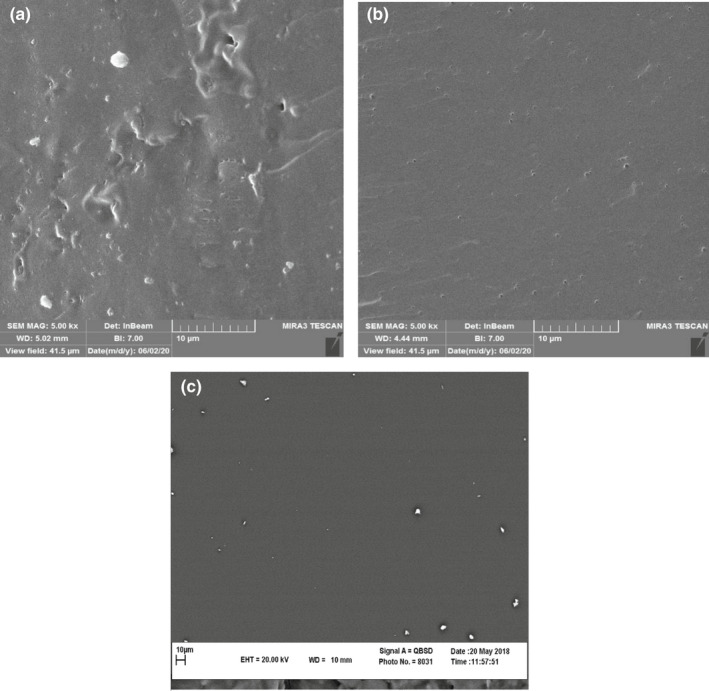
Scanning electron microscopy cross‐section images of films, Chicken feet extraction film (a), muscle fascia extraction film (b), and bovine bone gelatin film (c)

The films’ surface morphology was investigated by scanning electron microscopy (Figure 3). The results demonstrated that the cross‐linked Gel film was observed to be uniform, compact, and homogenous in appearance, while the microstructures of the MF film contained some bubbles. The surface of the CF film was rougher than MF and Gel films. The increase in surface roughness of CF film is attributed to the migration of fat droplets toward the film surface during the film drying process.

### 3.9 FTIR spectra of the films

**FIGURE 4:**
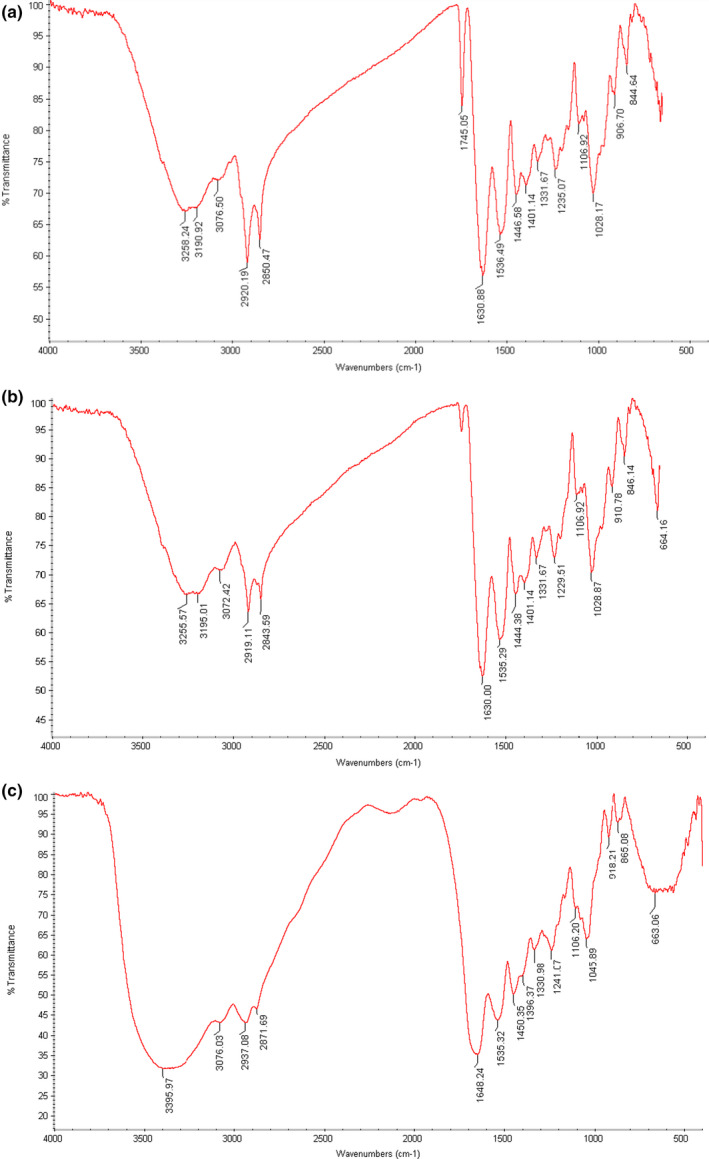
Fourier transform infrared spectroscopy (FT‐IR spectrum) of films. MF: Muscle fascia film (a), CF: chicken feet film (b), and Gel: bovine bone gelatin film (c)

FTIR spectroscopy has been extensively used for the identification of intermolecular interactions in polymers previously (Sionkowska, 2011; Liu et al., 2004). The properties of polymers are the result of interactions by hydrogen bonds and/or electrostatic interactions between the functional groups of the various polymers. Fourier transform infrared (FTIR) spectroscopy studies depicted bands formed by four individual peaks: amide A, amide I, amide III, and aliphatic alcohol. Figure 4a shows the FTIR spectra of the MF film. Moreover, Figure 4b shows the FTIR spectra for the CF and Gel films. The FTIR spectra of the MF film showed major peaks in the amide region. The MF film showed vibration peak at the wavenumbers of 1630.68 cm^‐1^ as the amide I, 1536.49 cm^‐1^ as the amide II, 1235.07 cm^‐1^ as the amide III, 2920.19 cm^‐1^ as the amide B, and of 3190.92 to 3258.24 cm^‐1^ as the amide A. Amide A peaks waxed more intensely, and both widened and sharpened with the increase in glycerol content in the films. This is likely due to the ‐OH group contributed by the plasticizer. The FTIR spectra of the CF film showed amide II at 1535.29 cm^‐1^, amide I at 1630.00 cm^‐1^, amide B at 2919.11 cm^‐1^, amide A in a range of 3195.01 to 3255.57 cm^‐1^, and amide III at 1229.51. The aliphatic alcohol group had glycerol content at a peak of 1028 cm^‐1^ for both types of films. Our data are similar to da Almeida and Lannes (2013) and Nor et al. (2017), who investigated the FTIR characterization of chicken feet gelatin and the effects of plasticizer concentrations on functional properties of chicken skin gelatin films, respectively (Nor et al., 2017; Almeida and Lannes, 2013). The spectra for Gel film showed that the band was formed by four individual peaks; situated at amide‐A, free water (3395.97 cm^‐1^), amide‐I (1648.24 cm^‐1^), amide‐II (1535.42 cm^‐1^), and amide‐III (1241.07 cm^‐1^). The peak situated around 1045 cm^‐1^ might be related to the possible interactions arising between plasticizer (OH group of glycerol) and film structure. These results were in line with previous studies (Hanani et al., 2019). Generally, similar spectra for the tree types of films were observed.

### 3.10 Differential scanning calorimeter (DSC)

Several phase transitions can occur when polymer materials are thermally processed, and each transition relates to a certain thermal property. The glass transition temperature (Tg), which is defined as “as the temperature at which a polymer undergoes a structural transition from a glassy to a rubbery state,” is one of these transitions. Under the Tg value, the films are rigid, stiff, and glassy, in comparison to levels greater than Tg, where the films become elastic and soft (Yang & Paulson, 2000). Additionally, there is the melting transition (Tm), where, at a temperature known as the melting temperature, there is a transition from a crystalline to an amorphous phase, a liquid‐like state (Alavi et al., 2014). The results showed only one Tg for the films (Table 2). Corresponding to Ghasemlou et al. (2011), if a plasticizer is not homogenized with a polymer, the mixture would have two Tg values, corresponding to two pure phases (Ghasemlou et al., 2011). The glass transition, melting, and deterioration peaks of the films prepared from CF, MF, and Gel films were around 33.80°C–35.42°C, 28.54°C–30.36°C, and 38.55°C–40.53°C, respectively. From the gelatin film, the Tg results reported may be explained by the block copolymer model for the amino acid content of gelatin. The Tg of gelatin takes place at ~60°C and is related to the Tg of its amino acid in the peptide chain. Additionally, native fish gelatin film had a Tm of 76.5°C, which is found to be higher than films produced from bovine skin gelatin, which have been shown to have a Tm of 65.06°C by De Carvalho and Grosso (2004) (De Carvalho & Grosso, 2004). Furthermore, the dry film derived from pigskin gelatin has been shown to have a Tm of 91°C (Bigi et al., 2001). The variation in observed thermal properties is appreciable due to different versions of the gelatin source used. Moreover, Bigi et al. (2004) described that the initial transition is likely correlated with the evaporation of absorbed water in the gelatin sample, and the higher temperature transition is associated with the relative quantity of the triple helix in gelatin. As such, thermal properties of films are influenced by their source (Bigi et al., 2004).

### 3.11 Radical scavenging activity of the films

There is a great deal of interest in food packaging today that offers antioxidant properties to prevent or delay lipid oxidation. The DPPH method is a widely used method to evaluate antioxidant activity. This method of analyzing the ability of compounds to behave as free radical scavengers or hydrogen donors was established on the ability of DPPH, a stable free radical, to be satiated, and therefore, decolorized when antioxidants are present. Consequently, there is a reduction in absorbance values (Siripatrawan & Harte, 2010). The result indicated that the MF film had no antioxidant activity, while DPPH scavenging activities of the CF film were 18.42% (Table 2) and 1.88% for the Gel film. Compared to edible films obtained from other sources, such as methyl‐cellulose (<5%; Noronha et al., 2014) and chitosan (<10% and 12%) (Moradi et al., 2012), the antioxidant activity of CF film is greater in comparison to other natural polymers and does not include antioxidant additives. As oxidative reactions result in a substantial food wastage, there is an expanding use of synthetic antioxidants. However, synthetic antioxidants have questionable impacts on health. Therefore, natural antioxidants may have a more favorable outlook because of their safer qualities. Of note, CF film contains natural antioxidants, and this is an unpatrolled feature of a polymer for use in food packaging.

### 3.12 Sensory analysis

**FIGURE 5:**
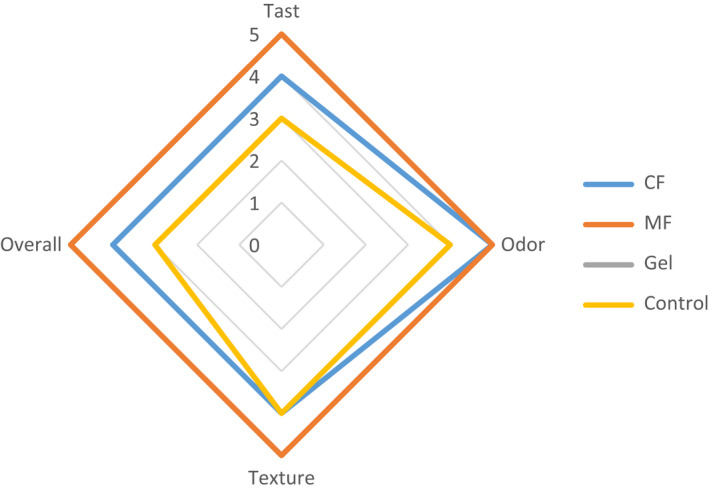
Sensory evaluation of films. MF: Muscle fascia film (a), CF: chicken feet film (b), and Gel: bovine bone gelatin film

**FIGURE 6:**
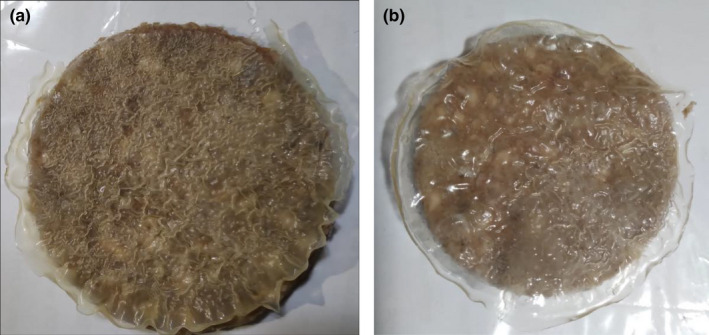
Burger covered with CF after defrosted (a), and Burger cover with MF after defrosted (b)

The result of the organoleptic evaluation of hamburger samples (control and wrapped) has been presented in Figure 5. Compared to the control samples, the burgers with film were easily separated after freezing. During defrost, films kept its shape and covered the burgers well (Figure 6). The appearance of the MF film was better than CF and gel on burgers. The MF film retained its clarity and uniformity while the CF and Gel films wrinkled during freezing. When cooking, the films were completely covered on the burgers. After cooking, the taste, odor, texture, and overall acceptance of burgers with MF and CF showed a higher score than the sample with Gel film and control.

## 4 CONCLUSION

Chicken feet and ovine muscle fascia extracts can be used as a precursor to edible films. Although the concentration of hyaluronic acid in CF was more than in the MF extract, the amount of hyaluronic acid in both extracts was significant. The CF film had better WVP and antioxidant properties than the MF and Gel films. The physical, thermal, and mechanical properties varied depending on the type of films source. It appears that the TS of the Gel film was more acceptable than the CF and MF films. FTIR results clearly highlighted intermolecular interactions between film components. These interactions are related to the presence of amine, hydroxyl, and/or carboxylic groups in the polysaccharides (hyaluronic acid) and collagen in the CF and MF films. Accordingly, the different mechanical and thermal properties of biopolymer films can be a result of hydrogen bonds between the reactive groups of components. From these results, it can be concluded that CF film might be an appropriate packaging material that may be applied in food products. Films were selected for potential separators of burgers. In relation to this preliminary study of CF and MF films for the utilization of separation materials for burger slices, promising results were obtained and further research is warranted. As separation material of cheese slices, the results are promising and deserve further investigation.

## CONFLICTS OF INTEREST

The authors declare no conflict of interests.
